# Research on the Physiological Mechanisms of Nitrogen in Alleviating Plant Drought Tolerance

**DOI:** 10.3390/plants14182928

**Published:** 2025-09-20

**Authors:** Xichao Sun, Qi Miao, Yingchen Gu, Lan Yang, Peng Wang

**Affiliations:** 1 Agro-Environmental Protection Institute, Ministry of Agriculture and Rural Affairs, Tianjin 300191, China; sunxichao@caas.cn; 2 Anhui Provincial Key Laboratory of Nutrient Cycling and Arable Land Conservation, Soil and Fertilizer Institute, Anhui Academy of Agricultural Sciences, Hefei 230031, China; miaoqibody@163.com; 3 Key Laboratory of Tobacco Biology and Processing, Ministry of Agriculture and Rural Affairs, Tobacco Research Institute, Chinese Academy of Agricultural Sciences, Qingdao 266101, China; gyczc201@163.com; 4 College of Resources, Hunan Agricultural University, Changsha 410128, China

**Keywords:** nitrogen, drought stress, water use efficiency, root architecture, biological nitrogen fixation

## Abstract

Drought represents a paramount constraint on global agricultural productivity, imposing severe limitations on crop yield and quality across diverse agroecosystems. Nitrogen (N), functioning as an indispensable macronutrient fundamental to plant architecture, metabolism, and stress acclimatization, exerts a pivotal influence in modulating plant resilience to water deficit. Substantial evidence accumulated in recent years underscores that optimal N nutrition significantly enhances plant adaptive capacity under drought by improving intrinsic water use efficiency (WUEi), optimizing photosynthetic performance, augmenting antioxidant defense systems, promoting advantageous root architectural modifications, and stabilizing biological N fixation (BNF) symbioses. This comprehensive review synthesizes current knowledge on the intricate physiological and molecular mechanisms underpinning N-mediated drought mitigation. We meticulously examine regulatory roles of N in water relations and hydraulic conductivity, photosynthetic apparatus protection and carbon assimilation efficiency, N metabolic flux and assimilation homeostasis, reactive oxygen species (ROS) scavenging and osmotic adjustment, root system development and resource foraging strategies, BNF system robustness under water stress, and the complex signaling networks integrating N and drought responses. Furthermore, we critically evaluate existing research consensus, identify persisting controversies and knowledge gaps, and delineate future research trajectories and translational challenges. The overarching objective is to furnish a robust theoretical foundation for devising precision N management strategies and advancing the breeding of drought-resilient, nutrient-efficient crop cultivars suited to arid and semi-arid regions facing escalating climate variability.

## 1. Introduction

Drought stress is a major abiotic constraint limiting global agricultural productivity, posing severe threats to crop yield and food security amid escalating climate variability [1,2,3]. Water deficit rapidly impairs key physiological processes, including photosynthesis, hydraulic conductance, and nutrient assimilation, disrupting the carbon–water–nitrogen (N) metabolic nexus and ultimately reducing crop performance [4,5,6,7].

N, beyond its fundamental role as a constituent of amino acids, nucleic acids, and chlorophyll, serves as a central signaling molecule that orchestrates plant responses to drought [8,9]. Evidence indicates that optimal N nutrition enhances drought tolerance through improved water use efficiency (WUE, biomass produced per unit water consumed), photosynthetic protection, antioxidant defense, root architectural modifications, and stabilization of biological N fixation (BNF) in legumes [10,11,12,13,14,15]. However, the effects of N are both dose- and form-dependent. For example, nitrate often enhances hydraulic conductivity through aquaporin (AQP) regulation, while excessive or ammonium-based fertilization may intensify stress responses [16,17]. Moreover, species-specific adaptations and interactions with concurrent stresses (e.g., heat, elevated CO_2_) further complicate N management strategies [18,19,20].

Despite advances, significant gaps remain in understanding the spatiotemporal dynamics, species-specific responses, and molecular mechanisms underlying N-mediated drought resilience. This review synthesizes current knowledge on the physiological and molecular mechanisms by which N alleviates drought stress, spanning water relations, photosynthesis, antioxidant systems, root architecture, BNF, and cross-stress interactions. We critically evaluate existing consensus, identify controversies, and propose future research directions to inform precision N management and breeding for drought-resilient, nutrient-efficient crops.

## 2. Physiological Mechanisms of N in Alleviating Plant Drought Stress

### 2.1. Regulation of Water Use and Photosynthesis by N Under Drought Stress

N exerts a tripartite influence-acting simultaneously as a signal, a structural component, and a nutrient-within the drought-stressed plant system. It serves as a primary substrate for the biosynthesis of photosynthetic machinery while crucially modulating the integrated “water uptake–translocation–consumption–carbon assimilation” continuum through its regulation of AQP and key photosynthetic enzyme complexes [21,22]. Under conditions of moderate water deficit, a predominance of nitrate in the root zone generally promotes the maintenance of root hydraulic conductivity (Lpr) and leaf hydraulic conductance (Kleaf), facilitating sustained root pressure and xylem sap flow to the canopy [23]. This hydraulic stability underpins the preservation of partial stomatal aperture, ensuring continued CO_2_ diffusion into the leaf mesophyll for carboxylation by Rubisco, thereby stabilizing photochemical efficiency (particularly PSII function, measured as variable fluorescence over maximum fluorescence (Fv/Fm)) and net photosynthetic rate (Pn) [24,25]. Consequently, intrinsic water use efficiency (WUEi, the ratio of photosynthetic carbon assimilation to stomatal conductance) and WUE often exhibit significant improvement under moderate NO_3_^−^ nutrition during drought [26].

This hydraulic–photosynthetic coupling effect facilitated by optimal N is demonstrable across phylogenetically diverse species. In the perennial grass *Leymus chinensis* subjected to moderate-severe drought, supplementation with NO_3_^−^ (5 mM) resulted in a 12% increase in Fv/Fm compared to N-deficient controls [27]. Similarly, *Catalpa bungei* seedlings exposed to vertical partial root-zone drying (V-PRD) stress achieved maximal WUEi when supplied with 1 mM N, concomitant with observable xylem vessel thickening, enhancing hydraulic efficiency [28]. Maize seedlings experiencing polyethylene glycol (PEG)-induced osmotic stress recovered key photosynthetic parameters (Pn, Fv/Fm) to 80–95% of well-watered control levels when nourished with 7.5 mM N, whereas both N deficiency and supra-optimal N exacerbated the stress damage, significantly impairing photosynthesis and growth [29]. Winter wheat under extreme soil water deficit (16% volumetric water content) coupled with high N fertilization exhibited a precipitous decline of approximately 50% in both N use efficiency (NUE) and WUE, performing markedly worse than plants receiving moderate N inputs [30]. The phenomenon of high N-induced hydraulic vulnerability underscores the counterproductive nature of excessive fertilization in water-limited environments (Table 1).

Furthermore, the chemical form of N significantly modulates plant drought tolerance trajectories. The apple rootstock *Malus prunifolia* exhibits a distinct physiological preference, markedly increasing NH_4_^+^ influx rates under PEG-simulated drought stress [31]. This shift, which aligns with its N-conserving strategy under drought, is consistent with the energy-efficient nature of NH_4_^+^ uptake. Unlike NO_3_^−^, which requires active transport via nitrate transporters (NRTs) and consumes ATP, ammonium enters passively through ammonium transporters (AMTs) without energy cost [32,33]. Transcriptionally, *Malus prunifolia* upregulates high-affinity AMTs (AMT1;2, AMT4;2) and downregulates NRTs, reducing energy expenditure under stress while maintaining cellular K^+^/Na^+^ balance [31,32]. This contrasts sharply with graminaceous species like maize and wheat, which rely on NO_3_^−^-induced AQP activity for hydraulic stability [23,29], highlighting species-specific N acquisition strategies.

**Table 1:** Physiological responses of different crops to N levels under drought conditions.

Crop Species	N Treatment	Key Physiological Changes	Reference
Maize (*Zea mays*)	7.5 mM N supply	Photosynthetic parameters (Pn, Fv/Fm) recovered to 80–95% of controls; H_2_O_2_ and O_2_^−^ levels reduced by over 40%	[29]
Winter Wheat (*Triticum aestivum*)	Moderate N supply (≈7 mM total N)	Activities of glutamine synthetase (GS1) and glutamate synthase (Fd-GOGAT) maintained at 80–90% of controls	[34]
*Catalpa bungei*	1 mM N + V-PRD	WUEi maximized; xylem vessel radial expansion enhanced hydraulic conductivity	[28]
*Leymus chinensis*	5 mM NO_3_^−^ supplementation	Fv/Fm increased by 12% compared to N-deficient groups	[27]
Winter wheat (*Triticum aestivum*)	High N treatment (≈30 g N m^−2^)	Stomatal conductance decreased; transpiration loss increased; yield reduced due to rapid soil moisture depletion	[35]
*Malus prunifolia*	1 mM NH_4_NO_3_	Net NH_4_^+^ influx increased; AMT1;2 and AMT4;2 up-regulated; biomass and photosynthesis higher than low-N drought group	[31]

### 2.2. Regulatory Mechanisms of N Signaling and N Assimilation Metabolism on Drought Stress

N form and AQP synergistically regulate hydraulic homeostasis and stomatal optimization. The rapid signaling interplay between N form and AQP regulation constitutes a frontline defense mechanism against early drought. Nitrate acts as a potent signal, inducing within 1–3 h the transient transcriptional upregulation and possible post-translational activation (e.g., via phosphorylation) of specific plasma membrane intrinsic proteins (PIPs), notably PIP1;2 and PIP2;1 isoforms [36,37]. This rapid response significantly enhances Lpr and Kleaf [38,39]. Enhanced hydraulic conductance allows plants to maintain partial stomatal opening during the initial stages of drought, preserving CO_2_ influx for photosynthesis while minimizing water loss—a delicate balance critical for early stress acclimation [40]. This dual benefit of water conservation and photoprotection highlights the signaling role of NO_3_^−^ beyond nutrition.

The integration of N and drought signaling pathways involves key regulatory nodes. In *Arabidopsis thaliana*, the calcium-dependent protein kinase CPK6 is transcriptionally up-regulated by abscisic acid (ABA) accumulation under the combined stress of high nitrate (10 mM NO_3_^−^) and drought [41]. Activated CPK6 phosphorylates the dual-affinity nitrate transporter NRT1.1 (CHL1) at a specific threonine residue (Thr101 in *Arabidopsis*; equivalent to Thr571 in some reports). This phosphorylation event specifically inhibits NRT1.1′s low-affinity nitrate transport activity without affecting its high-affinity function [42]. This molecular switch effectively reduces nitrate influx under stress conditions, contributing to a decrease in transpiration rate (likely linked to reduce osmoticum accumulation or altered stomatal signaling) while ensuring a sustained, albeit modulated, supply of nitrate to the shoots. This coordinated response enhances overall survival under severe drought, exemplifying a genetically targetable water–N crosstalk pathway amenable to manipulation for improved stress tolerance [43].

N supply optimizes metabolic flux, conserving energy and redirecting reductive power. Drought stress necessitates a profound reorganization of primary metabolism, and N assimilation is central to this reprogramming. The rice (*Oryza sativa*) C_2_H_2_-type zinc finger transcription factor drought and salt tolerance (DST) serves as a critical regulator at the nexus of N metabolism and drought response. Under well-watered conditions, DST directly activates the expression of *OsNR1.2*, encoding a major nitrate reductase (NR) isoform, promoting the reduction of NO_3_^−^ to NO_2_^−^. However, upon drought perception, DST is rapidly transcriptionally repressed or post-translationally inactivated. This leads to a significant downregulation (up to 65%) of *OsNR1.2* transcript levels, drastically slowing the conversion of NO_3_^−^ to NO_2_^−^ [44]. This strategic metabolic throttling conserves substantial amounts of reductant (NAD(P)H) and ATP that would otherwise be consumed by NR and subsequent nitrite reduction steps.

Maintaining the glutamine synthetase/glutamate synthase (GS/GOGAT) cycle homeostasis is critical under drought. While NR activity is often suppressed, sustaining GS/GOGAT cycle is paramount for incorporating ammonium into amino acids and preventing toxic NH_4_^+^ buildup, especially water stress [45]. Studies on winter wheat revealed that under moderate N supply (≈7 mM total N), even when drought significantly inhibited NR activity, the combined activities of cytosolic GS1 and chloroplastic Fd-GOGAT remained remarkably resilient, maintaining 80–90% of their activity levels observed in unstressed controls [34]. This metabolic stability ensures the continued assimilation of NH_4_^+^ (whether derived from residual NR activity, photorespiration, or protein turnover) into glutamine and glutamate, the primary amino donors for biosynthesis. Sustained GS/GOGAT flux prevents a critical metabolic imbalance: the uncoupling of carbon fixation (which may continue at reduced rates) from N assimilation [46]. Such uncoupling would lead to the deleterious accumulation of soluble sugars (e.g., sucrose, hexoses) and compatible solutes (e.g., proline, glycine betaine) to osmotically detrimental levels, exacerbating cellular dehydration and potentially triggering feedback inhibition of photosynthesis or premature senescence [47,48].

### 2.3. Antioxidant and Osmotic Regulation Mechanisms Mediated by N Under Drought

Drought-induced cellular dehydration is invariably accompanied by a massive overproduction of reactive oxygen species (ROS), including superoxide anion (O_2_•^−^), hydrogen peroxide (H_2_O_2_), and hydroxyl radicals (•OH), primarily originating from impaired photosynthetic electron transport in chloroplasts and enhanced respiratory activity in mitochondria [49,50]. Accumulated ROS inflict severe oxidative damage on cellular components-oxidizing lipids (membrane disintegration), proteins (enzyme inactivation, carbonylation), and nucleic acids (mutations, strand breaks) ultimately leading to cell death and organ dysfunction [51]. Optimal N nutrition serves as a crucial metabolic buffer under these conditions, modulating the plant’s capacity to manage ROS levels and maintain cellular osmotic potential (Ψs), thereby bolstering cellular integrity, membrane stability, and overall drought tolerance [52,53]. This regulation typically exhibits a distinct threshold or optimum.

For instance, sunflower (*Helianthus annuus*) plants subjected to drought stress displayed a synchronized increase in the activities of key antioxidant enzymes-superoxide dismutase (SOD), catalase (CAT), and peroxidase (POD) within the N application range of 80–120 kg N ha^−1^ [54]. This enhanced enzymatic defense correlated with significant physiological recovery: leaf relative water content (RWC) and Pn reached 90% and 85% of their well-watered, N-sufficient control values, respectively. However, exceeding this optimal range (>150 kg N ha^−1^) proved detrimental, as N surplus triggered a secondary oxidative burst, likely due to metabolic imbalance, increased respiratory load, or photorespiratory H_2_O_2_ overproduction, negating the initial protective effects and exacerbating stress symptoms [54]. Similarly, drought-stressed maize seedlings supplied with 7.5 mM N exhibited a 1.8-fold increase in NR activity and tissue NO_3_^−^ concentration, while concurrently reducing H_2_O_2_ and O_2_•^−^ levels by over 40% compared to N-deficient stressed plants [29]. This highlights the role of N in both boosting N status and enhancing ROS scavenging capacity (Table 2).

N actively primes the antioxidant defense system. Drought-stressed cotton seedlings receiving 5 mM N showed pronounced upregulation of ascorbate peroxidase (APX) and glutathione reductase (GR) activities, alongside increased accumulation of the non-enzymatic antioxidants reduced glutathione (GSH) and ascorbate (ASA). This integrated response culminated in malondialdehyde (MDA) levels being reduced to only 55% of those measured in drought-stressed, low-N plants, indicating significantly less membrane damage [55]. N also profoundly influences osmotic adjustment, a key mechanism for maintaining cell turgor and hydration under water deficit. In maize subjected to severe PEG-induced drought combined with 180 kg N ha^−1^ fertilization, the enzymatic pathways for proline synthesis, namely Δ^1^-pyrroline-5-carboxylate synthetase (P5CS) and ornithine aminotransferase (OAT), were significantly activated. Consequently, proline accumulation increased by 24% compared to low-N stressed plants. Proline serves a dual function, acting as a potent compatible osmolyte to lower cellular osmotic potential and retain water, and serving as a direct scavenger of •OH radicals, thus protecting protein structure and membrane integrity [56]. Wheat plants under deficit irrigation (60% of crop evapotranspiration) supplemented with 0.6 g N kg^−1^ soil showed a 30% increase in soluble sugars (e.g., sucrose, fructans) and a 22% increase in soluble proteins, alongside a 28% reduction in electrolyte leakage (a measure of membrane damage) [57]. This demonstrates capacity of N to remodel carbon–N metabolism, promoting the accumulation of osmotically active compounds that contribute to cellular turgor maintenance and membrane stabilization under dehydration stress.

Notably, N-mediated antioxidant and osmotic regulation often synergizes with potassium (K), a primary counterion of NO_3_^−^ during root uptake [58]. K maintains vacuolar nitrate storage and cytoplasmic ion homeostasis, thereby indirectly enhancing N’s role in osmotic adjustment. For example, in maize, the combined application of N and K increased proline accumulation compared to N alone, while also reducing H_2_O_2_ levels through enhanced SOD activity [59]. K also stabilizes the ascorbate–glutathione cycle by maintaining GR enzyme structure, which amplifies N-induced APX activity [60]. This N-K synergy is critical for avoiding ionic imbalances (e.g., Na^+^ overaccumulation) under drought, as NO_3_^−^ and K^+^ co-transport reduces the risk of toxic cation uptake. Thus, neglecting K supply may limit the efficacy of N-based drought mitigation strategies.

Beyond biochemical adjustments, N influences structural components that enhance hydraulic and mechanical resilience [52]. Studies demonstrated that wheat grown under deficit irrigation and supplied with 0.6 g N kg^−1^ soil developed significantly reinforced vascular and sclerenchyma tissues. Specifically, xylem vessel diameters increased by 18%, enhancing hydraulic conductivity under tension, while the thickness of sclerenchyma fibers surrounding vascular bundles increased by 25% [57]. This structural reinforcement improves mechanical strength and, critically, helps prevent or delay the propagation of drought-induced xylem cavitation (air embolisms), which can severely disrupt water transport [61,62].

Despite consistent evidence that moderate N enhances antioxidant activity, controversies persist regarding the thresholds for excessive application. Research has shown that secondary oxidative stress can occur in sunflower at N levels exceeding 150 kg N ha^−1^ [54]. In contrast, other studies have found no such effect in cotton even at applications above 200 kg N ha^−1^ [55]. This discrepancy may stem from crop-specific ROS scavenging capacity or soil water holding capacity. Additionally, most studies are based on single drought events, whereas long-term intermittent drought may alter the role of N. Research indicates that species such as *Leymus chinensis* required approximately 20% more N to maintain Fv/Fm under repeated drought conditions, which can be attributed to cumulative N losses through leaching [27]. These gaps highlight the need for crop-soil-climate specific N recommendations.

### 2.4. N-Mediated Root Morphology and Optimized Resource Acquisition Under Drought Stress

Root system architecture (RSA) is a primary determinant of a plant’s ability to forage for essential soil resources, particularly water and N, under limiting conditions [63,64]. N availability acts as a powerful morphogenic signal that dynamically reshapes RSA in response to drought. This process manifests through three predominant, often contrasting patterns: moderate N in the upper layer induces deeper root penetration into subsoil strata; low N promotes fine root proliferation to enhance absorptive surface area within explored soil volumes [65]; and high N favors shallow rooting, restricting exploration predominantly to surface layers while often reducing overall root investment relative to shoots [66]. Both extremes compromise the plant’s drought adaptability: excessive N triggers rapid depletion of surface soil moisture through intensified transpiration, whereas N deficiency restricts access to deep soil water due to insufficient root biomass. In contrast, moderate N levels achieve an optimal balance between deep root growth and fine root density, enabling maximal utilization of both deep soil moisture and nitrate. This demonstrates that suboptimal N levels (i.e., not the highest levels)—relative to well-watered conditions—actually enhance drought-induced root plasticity [67].

Pot experiments simulating Northeast China’s rainfed conditions for maize clearly demonstrated the benefits of moderate N under drought. A 30% reduction in N application (simulating moderate N input) under concomitant moderate drought stress stimulated a 30–50% increase in root length density (RLD) specifically in soil layers below 40 cm depth. Critically, fine root RLD (diameter < 0.5 mm) reached 0.30 cm^−3^ in these deeper layers [68]. This architectural remodeling involves sophisticated signaling, which is as follows: perception of localized NO_3_^−^ gradients, auxin (IAA) redistribution directing growth towards N-rich patches, and activation of the sucrose non-fermenting-1-related kinase 1 (SnRK1) energy-sensing pathway which influences carbon allocation and sugar transporter expression, facilitating carbon export to support root growth in resource-rich zones [69,70]. The resulting deep root column phenotype, with enhanced lateral root branching, significantly improved access to deep water, boosting WUE to 4.1 g L^−1^, and enhanced nitrate capture efficiency, increasing NUE by 55% compared to conventional high-N management [68] (Table 3). In stark contrast, maize subjected to extreme drought combined with complete N deprivation exhibited a passive survival strategy. While the taproot might elongate somewhat in search of water, total root biomass plummeted, and the specific N uptake rate per unit root length dropped to merely one-third of that observed under adequate N, resulting in the poorest resource capture efficiency and minimal growth [71].

High N fertilization consistently induces a shallow root system phenotype, characterized by reduced root depth exploration, a lower root-to-shoot biomass ratio (R/S), and often excessive shoot growth (leaf area expansion). This root system architecture is inherently vulnerable under drought conditions for three key reasons. First, shallow roots predominantly access surface soil layers that dry most rapidly. Second, high N elevates canopy transpirational demand by increasing leaf area index (LAI). Finally, deep soil nitrate reserves remain underutilized due to insufficient root exploration. Consequently, high-N plants develop disproportionate vulnerability to water stress. This manifests through elevated evapotranspiration accelerating soil moisture depletion, inefficient deep N utilization, and ultimately significant reductions in both WUE and NUE [72,73,74]. This vulnerability is amplified when drought occurs post-anthesis in cereals relying on remobilized N from deeper roots [75,76].

The principle extends beyond annual crops to woody perennials. *Catalpa bungei* seedlings supplied with sufficient N and exposed to V-PRD where only part of the root system is watered-activated a synergistic response: proliferation of fine roots in the moist zone and radial expansion of xylem vessels [77]. The synergistic effect of deep root exploration and large vessel conductance substantially improved hydraulic efficiency and drought survival rates in seedlings, outperforming those subjected to uniform drought or inadequate N supply [78]. In grassland ecosystems, functional diversity dictates species-specific responses to N addition under drought. Shallow-rooted grass species (e.g., *Lolium perenne*) often show a positive yield response to top-dressing with moderate N (e.g., 60 kg N ha^−1^) during severe drought, potentially compensating for stress-induced growth reduction and promoting post-drought regrowth [79]. Deep-rooted N-fixing legumes such as *Medicago sativa* contrastingly demonstrate minimal responsiveness to mineral N supplementation. Their dependence on symbiotic N fixation and subsoil water resources largely decouples them from soil mineral N pools while deep roots simultaneously buffer water stress particularly during drought [80,81].

Rational agronomic management prioritizing balanced N-water regimes—exemplified by regulated deficit irrigation (RDI) or partial root-zone drying (PRD)—harnesses inherent RSA plasticity [82,83]. This strategy activates the nitrate gradient-NRT1.1 auxin signaling pathway [84,85] while promoting development of synergistic deep-rooted frameworks integrated with dense fine root networks. Concurrent integration with the PIP-NRT2-nitric oxide metabolic system [86,87,88] enables synchronous capture of deep soil water and nitrate resources. Consequently this approach establishes viable pathways for dual enhancement of agricultural water-use and N-use efficiencies within arid and semi-arid ecosystems [89,90].

Notably, the optimal N concentration for root morphological adaptation (e.g., deep rooting in maize: 30% reduced N input [68]) is lower than that for antioxidant/osmotic defense (e.g., sunflower: 80–120 kg N ha^−1^ [54]) or photosynthesis maintenance (maize: 7.5 mM [29]). This difference occurs because root proliferation under low N conditions prioritizes resource foraging through energy allocation to roots. In contrast, antioxidant defense and photosynthesis require higher N inputs for the synthesis of key enzymes such as SOD and Rubisco. These distinct N demands underscore the importance of stage-specific N management—for instance, limiting N supply during the seedling stage to promote root development, while providing moderate N during flowering to support defense mechanisms.

### 2.5. Drought Adaptation Mechanisms of BNF Systems

BNF, the enzymatic reduction of atmospheric N_2_ to ammonia by rhizobia symbiotically housed within legume root nodules, is an energy-intensive process highly sensitive to environmental perturbations, particularly drought. Drought stress rapidly compromises BNF through two interconnected mechanisms. First soil desiccation reduces nodule water potential, diminishing bacteroid hydration and metabolic activity. Second impaired canopy photosynthesis restricts sucrose supply to nodules limiting energy provision for nitrogenase function and bacteroid respiration [91,92]. Consequently, Nase enzyme activity (commonly monitored via the *nifH* gene product) and the concentration of oxygen-binding leghaemoglobin (essential for maintaining the microaerobic conditions required for Nase function) decline precipitously [93] (Table 4). Critically, BNF inhibition often manifests before significant reductions in whole-plant photosynthesis become apparent, making it one of the most drought-sensitive components of legume physiology [91,94]. Split-root experiments with pea (*Pisum sativum*) provided elegant proof that this inhibition is primarily localized: nodules on roots within the drying soil compartment exhibited a sharp decline in N fixation rate, while nodules on roots in the well-watered compartment maintained near-normal activity. This demonstrates that the inhibition stems directly from the localized drop in water potential and/or carbon supply within the affected root zone, rather than being solely governed by systemic whole-plant N status feedback signals [95]. Addressing this localized nodule inactivation bottleneck requires integrated management strategies targeting the nodule microenvironment and whole-plant carbon economy.

Co-inoculation with osmotolerant *Bradyrhizobium elkanii* and *Azospirillum brasilense* (a plant growth-promoting rhizobacteria (PGPR)) enhances soybean drought tolerance by stabilizing *nifH* expression and Nase activity [96,97]. Under normal water conditions, this co-inoculation increases nodule number and grain yield compared to single *Bradyrhizobium elkanii* inoculation, with no significant competition—likely due to niche differentiation (*Azospirillum brasilense* colonizes rhizosphere, *Bradyrhizobium elkanii* forms nodules) [98,99]. Cost-wise, the additional PGPR inoculant increases input costs, but field trials in Brazil show more return on investment via yield gains [100]. However, efficacy declines in high-fertility soils, as excess mineral N suppresses nodulation [101].

Deployment of slow-wilting soybean genotypes combined with water conservation management confers inherent physiological advantages. This approach leverages cultivars demonstrating delayed canopy wilting phenotypes under water deficit conditions typically associated with deeper rooting systems or conservative water-use strategies [96]. Combining these genotypes with soil management practices that enhance water retention is synergistic. No-till farming and organic amendments (e.g., cover crops) enhance soil water-holding capacity, reducing nodule inactivation by 30–40% under drought and sustaining carbon supply to nodules for BNF [102,103,104].

Strategic supplemental N fertilization can be effective during severe drought coinciding with peak crop demand periods such as soybean pod filling. At this physiological stage BNF suppression creates substantial N deficits inadequately compensated by soil mineralization or residual soil N. Applying a moderate supplemental dose (40–60 kg N ha^−1^) of a balanced N source like NH_4_NO_3_ during the most acute drought phase can temporarily bridge this N gap. Field studies indicate this strategy can increase final soybean yield by 15–25% under severe drought compared to relying solely on compromised BNF [105,106]. However, precision is paramount: higher application rates (>80 kg N ha^−1^) typically offer no additional yield benefit and can be detrimental. Excessive mineral N especially ammonium ions suppresses residual BNF activity while inhibiting long-term nodule formation and function. More critically it stimulates disproportionate vegetative growth and transpiration thereby exacerbating plant water stress and potentially negating yield gains [4,101].

This synergistic strategy integrating drought-adapted rhizobia delayed-canopy-wilting genotypes water-conserving management and minimal supplemental N establishes multi-tiered drought resilience. The approach functions across three spatial scales: nodule-level osmoprotection enhancing bacteroid resilience rhizosphere-scale moisture retention improving soil structure and field-level canopy water conservation coupled with precision N supplementation. Together these buffers significantly mitigate drought impacts on symbiotic N fixation systems creating robust physiological foundations for stable soybean yield and other essential legume crops in drought-vulnerable agroecosystems [107,108].

While co-inoculation with osmotolerant rhizobia enhances BNF under drought conditions, its effectiveness depends on soil pH. Research shows that the resulting yield increase is significantly higher in acidic soils compared to alkaline environments, which may be attributed to poorer rhizobial survival under alkaline conditions [96,109]. Additionally, supplemental N during pod filling may suppress residual BNF in some cultivars (e.g., Williams 82 soybean) [110], indicating genetic variation in BNF sensitivity to mineral N. These inconsistencies emphasize the need for integrated genotype-microbe-N management.

**Table 4:** Strategies for maintaining BNF in legumes under drought.

Regulation Strategy	Technical Details	Effects Under Drought Conditions	Reference
Inoculation with drought-tolerant rhizobia and PGPR	Co-inoculation with osmotic stress-tolerant rhizobia (e.g., *Bradyrhizobium elkanii*) and PGPR (e.g., *Azospirillum brasilense*)	Stabilized *nifH* gene expression and Nase activity; soybean yield increased by 15–25% compared to single rhizobia inoculation	[96]
Water-retentive soil management	No-till combined with organic amendments to enhance soil organic matter (SOM) and water-holding capacity	Reduced nodule inactivation by 30–40%; sustained carbon supply to nodules; alleviated BNF inhibition	[104]
Use of slow-wilting genotypes	Selection of deep-rooted or water-conservative cultivars; delayed canopy wilting under water deficit	Mitigated drought impacts on nodule water potential; maintained BNF function until grain filling	[96]
Targeted N supplementation	Application of 40–60 kg N ha^−1^ NH_4_NO_3_ during critical drought stages (e.g., soybean pod filling); avoidance of excessive N to prevent BNF suppression	Temporarily compensated for reduced BNF; yield increased by 15–25% vs. sole reliance on BNF; high N (>80 kg N ha^−1^) ineffective or harmful	[105]

### 2.6. N Regulation Strategies Under Multiple Stress Scenarios

Climate change is driving an increase in the frequency and intensity of compound abiotic stress events, where plants simultaneously or sequentially experience combinations like high temperature (heat), drought, and elevated atmospheric CO_2_ concentrations [111,112]. Under complex drought scenarios simplistic N maximization paradigms prove obsolete requiring nuanced fertilizer management. N application functions as a dual-effect tool demanding rigorous optimization to prevent stress amplification while maintaining productivity [113]. Research on winter wheat experiencing concurrent heat stress (36/26 °C day/night) and soil water deficit (45–55% field capacity) during the sensitive jointing stage revealed that a medium N application rate (≈21 g N m^−2^) outperformed the traditional high N rate (≈30 g N m^−2^). Medium N better sustained stomatal conductance and modulated instantaneous transpiration, allowing Pn and WUE to increase synchronously by 15–20% compared to high N. The high N treatment amplified water loss through excessive transpiration, ultimately reducing grain yield due to accelerated soil moisture depletion and possible heat damage under constrained water supply [35].

The interaction with elevated CO_2_ adds another layer of complexity. While CO_2_ enrichment (e.g., 800 ppm) typically stimulates Pn and improves WUE in C3 plants by reducing photorespiration and stomatal conductance, it simultaneously induces leaf carbon-to-N (C:N) dilution [114,115]. This dilution occurs because the increased carbon assimilation outpaces N uptake and assimilation, leading to a relative N deficiency that can constrain protein synthesis and growth [116]. Studies on tomato (*Solanum lycopersicum*) and barley (*Hordeum vulgare*) grown under 800 ppm CO_2_ confirmed that moderate, split N applications (100–200 kg N ha^−1^ total) effectively compensated for CO_2_ induced C:N dilution, supporting leaf N concentration and protein content while avoiding excessive transpiration [18].

N chemical form also exhibits significant combination buffering effects under stress. Experiments simulating atmospheric N deposition on wheat showed that depositing only nitrate or only ammonium during mid-to-late growth stages depressed Pn compared to background conditions. However, depositing a mixture of NO_3_^−^, NH_4_^+^, and urea (mimicking a more realistic, complex N deposition scenario) mitigated this photosynthetic decline [117]. The mixed N source likely provided greater metabolic flexibility, buffered rhizosphere pH fluctuations, and reduced ion-specific toxicity risks associated with single N forms under stress [33].

Furthermore precise alignment of N application timing with critical crop developmental stages termed the window effect determines efficacy under multifactorial stress conditions. Research on barley demonstrated that supplying moderate N during the tillering stage significantly increased tiller number and established a higher yield potential. However, maintaining high N availability into the later grain-filling stages under terminal drought proved detrimental. The high N status forced stomatal closure earlier and more severely as soil moisture declined, impaired the remobilization of stem reserves to grains, and resulted in significant reductions in both WUE and thousand-kernel weight [118]. This validation confirms preemptive N allocation with reproductive-phase restriction as an effective staged management strategy. Early vegetative N investment establishes canopy architecture and yield potential while subsequent N restriction during reproductive stages promotes conservative water utilization and efficient nutrient remobilization under projected drought stress [119].

Synthesizing empirical evidence from diverse crops and stress scenarios, the following refined N management principles emerge for multi-stress environments. To enhance resilience and productivity stability under increasingly variable and stressful climatic conditions, adhere to these N management principles. Under combined heat and drought stress, reduce total N application by 20–30% relative to standard practice, using split applications, to balance photosynthetic carbon gain with transpirational water loss and mitigate heat-enhanced sensitivity to ammonium. Under elevated CO_2_ with drought, employ moderate total N inputs, such as 80–120% optimal N dose adjusted for crop and soil, delivered in split applications to counteract carbon-to-N dilution while supporting sustained protein synthesis; avoid single large doses that stimulate excessive transpiration. Replace uniform high-N regimes with dynamic stage-optimized strategies, prioritizing N supply during tillering and vegetative establishment to build yield potential while consciously restricting N availability during sensitive reproductive stages, especially under drought risk, to promote water conservation, remobilization efficiency, and grain filling. Utilize blended N sources where possible for enhanced buffering capacity. This approach facilitates the concurrent optimization of photosynthetic performance, WUE, and NUE [120].

### 2.7. Molecular Regulatory Mechanisms and Breeding Targets

Advances in multi-omics technologies (genomics, transcriptomics, proteomics, metabolomics) and functional genomics have begun to unravel the intricate molecular circuitry connecting N sensing, hydraulic regulation, metabolic reprogramming, and the manifestation of drought tolerance phenotypes [121,122]. This knowledge provides a blueprint for targeted genetic improvement.

The DST-NR node, involving the C_2_H_2_ zinc finger transcription factor DST in rice, functions in energy conservation and ROS management by directly binding to the *OsNR1.2* promoter to activate its expression under favorable conditions, thereby driving nitrate reduction. Drought stress triggers rapid suppression of the DST-NR axis (via transcriptional down-regulation and/or post-translational modification), leading to a sharp decline in NR activity [44]. This strategic down-regulation conserves substantial pools of NAD(P)H and ATP. The conserved energy is redirected towards sustaining vital antioxidant pathways (e.g., ascorbate–glutathione cycle) essential for detoxifying drought-induced ROS [123,124]. Furthermore, limiting NR activity reduces the risk of toxic nitrite accumulation and minimizes over-reduction of the photosynthetic electron transport chain, a major source of ROS under stress [125]. Consequently, DST emerges as a high-priority molecular target for simultaneously enhancing drought survival and NUE. Allelic variants of DST or its orthologs associated with moderate NR suppression under stress offer significant potential for breeding [126].

Nitrate acts as a potent signal rapidly inducing the expression of specific *PIPs*, notably *PIP1;2* and *PIP2;1*, thereby enhancing AQP function for improved hydraulic efficiency. This upregulation enhances Lpr and Kleaf, facilitating water movement under drought and helping maintain stomatal conductance for CO_2_ uptake [36,37,127]. In the woody species *Catalpa bungei*, this PIP-mediated hydraulic enhancement synergizes with drought-induced xylem vessel enlargement to form a highly efficient deep root-large vessel water acquisition and transport system [28]. Engineering crops with enhanced *PIP* expression or stability, particularly in roots and vascular tissues, is a potential strategy for improving drought tolerance through better water management [128].

Maintaining high flux through the GS/GOGAT cycle is crucial for carbon-N homeostasis under drought, enabling effective ammonium assimilation, prevention of NH_4_^+^ toxicity, and sustained amino acid production for growth and stress protection [129]. Key enzymes include cytosolic GS1 (especially GS1;2 isoforms) and chloroplastic Fd-GOGAT. Alleles associated with sustained high activity of these enzymes under combined N and water stress have been identified in germplasm screens of wheat and maize [130,131]. Introgressing these favorable alleles into elite lines has shown potential for improving grain yield and NUE under drought, likely by preventing carbon-N imbalance and associated metabolic dysfunction [132].

Systems biology approaches integrating transcriptomics across developmental stages and stress treatments have identified core co-expressed gene networks associated with deep rooting and high NUE for QTL pyramiding. These loci show strong statistical association with traits like deep root mass, deep water extraction capacity, and high NUE in field trials. They are prime candidates for CRISPR-Cas9 mediated gene editing and for marker-assisted pyramiding in breeding programs [133,134]. Proof-of-concept was demonstrated in wheat (*Triticum aestivum*), where transgenic lines simultaneously overexpressing the AQP *TaPIP2;1* under a stress-inducible promoter and suppressing *TaDST* expression via RNAi or CRISPR showed synergistic improvements. These lines exhibited significantly increased WUE and NUE under controlled drought stress compared to wild-type or single-gene modified plants [135], validating the feasibility and potential of morpho-physiological dual-target editing strategies.

In summary, the convergence of multiple molecular pathways-spanning DST-mediated N metabolic economy, AQP-facilitated hydraulic efficiency, GS/GOGAT-driven assimilation stability, and QTLs governing deep rooting and efficient N utilization-alongside natural allelic variations discovered through genomics, delineates a suite of high-value target regions for molecular breeding. Harnessing these targets through advanced gene editing marker-assisted selection and genomic prediction offers strong potential for developing less N-demanding more drought-resilient crop varieties thereby establishing a crucial molecular foundation for climate-smart agriculture [136].

## 3. Conclusions

This review synthesizes compelling evidence that judicious N management, characterized by moderate total inputs corresponding to 70–90% of the optimal N dose and optimization of N form, activates a multifaceted suite of physiological and molecular mechanisms that collectively enhance plant resilience to drought stress. In most crops, nitrate is the preferred form, although ammonium may be more beneficial in certain species with specific physiological adaptations. These mechanisms operate across multiple organizational scales to improve WUE, NUE, and overall drought adaptability.

At the molecular level, nitrate acts as a rapid signaling molecule that upregulates the expression of *PIPs*, such as *PIP1;2* and *PIP2;1*, thereby enhancing Lpr and Kleaf. Concurrently, drought-induced suppression of the DST–NR axis conserves energy and reductant actions, redirecting resources toward ROS detoxification. Maintenance of the GS/GOGAT cycle ensures continuous assimilation of ammonium into amino acids, preventing NH_4_^+^ toxicity and metabolic uncoupling of carbon and N metabolism.

At the cellular levels, optimal N availability enhances the antioxidant defense system, elevating the activities of enzymes such as SOD, CAT, APX and GR, alongside increasing non-enzymatic antioxidants (ascorbate and glutathione). It also stimulates the biosynthesis and accumulation of key osmolytes, such as proline, soluble sugars including sucrose and fructans, and glycine betaine, all of which contribute to osmotic adjustment and membrane stabilization.

At the organ levels, moderate N promotes advantageous morphological plasticity within the root system, encouraging deeper rooting (mediated by auxin signaling, nitrate gradients, and DRO1-like genes) and fine root proliferation, significantly improving access to deep soil water and nitrate reserves. Structurally, N reinforcement of xylem vessels and sclerenchyma tissues enhances hydraulic efficiency and reduces cavitation risk.

At the symbiotic system level, co-inoculation with osmotolerant rhizobia (e.g., *Bradyrhizobium elkanii*) and PGPR (e.g., *Azospirillum brasilense*), combined with soil conservation practices (e.g., no-till farming, organic amendments), improves nodule tolerance to low water potential and helps sustain carbon supply, partially maintaining BNF under drought. Strategic, low-dose supplemental N application (50–70% of optimal NH_4_NO_3_) during peak drought can temporarily compensate for suppressed BNF, protecting yield.

**Figure 1:**
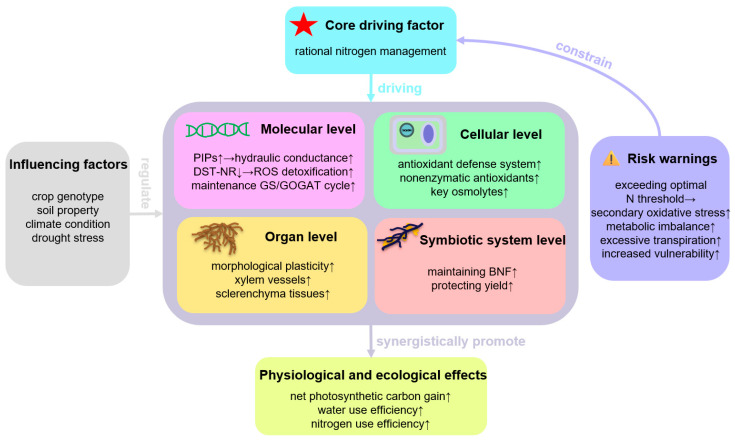
A schematic diagram of the multi-level regulatory mechanisms by which rational N management enhances plant drought resistance.

The synergistic operation of these mechanisms enhances net photosynthetic carbon gain, improves WUE, and boosts NUE. However, the efficacy of N-mediated drought mitigation is highly dependent on interactions among crop genotype, soil properties (e.g., texture, organic matter, inherent fertility), climate conditions (e.g., temperature, radiation, humidity), and the timing, intensity, and duration of drought stress. Crucially, exceeding the optimal N threshold can reverse benefits, inducing secondary oxidative stress, metabolic imbalance, excessive transpiration, and increased vulnerability to other stresses (Figure 1).

## 4. Prospects

Future research should prioritize closing critical knowledge gaps and developing translational solutions to optimize N management for drought resilience across diverse agroecosystems. Key directions involve advancing real-time precision N management by leveraging remote sensing, proximal sensors, and mechanistic crop models integrated with machine learning to dynamically define spatially and temporally variable optimal N windows under current and predicted drought conditions, while also developing user-friendly decision support systems for farmers [137,138,139]. Future efforts should prioritize multi-scale digital integration using digital twin platforms capable of simulating N–drought interactions across biological and agronomic levels, ranging from subcellular processes to whole-canopy dynamics. Such integration will accelerate hypothesis testing and support the optimization of management strategies through in silico modeling [140]. Additionally, integrated soil–plant–microbe management approaches are essential, combining high-throughput screening of drought-adapted microbes with region-specific conservation tillage and organic matter management, validated through long-term multi-location field trials to generate locally adapted guidelines [141,142]. Finally, precision molecular breeding should utilize high-resolution genomics and genome editing tools such as CRISPR-Cas to target key genes involved in N-enhanced drought tolerance, while employing machine learning-based genomic prediction to accelerate the development of resilient elite cultivars [143,144,145].

The ultimate goal is the co-development and deployment of an integrated technological framework encompassing precision N application (based on dynamic percentage regulation of the local optimal N dose), microbial symbiont synergy, advanced molecular breeding, and AI-driven intelligent decision support. This holistic approach may contribute to the transition toward high-yielding, resource-efficient (low N, high water productivity), and climate-resilient agricultural systems, essential for ensuring sustainable food security in the face of escalating water scarcity and climate volatility in arid and semi-arid regions worldwide.

## Figures and Tables

**Table 2:** Responses of plant antioxidant and osmotic regulation systems under N regulation.

Regulation Type	Key Substance/Enzyme	Effects of Moderate N Treatment	Potential Risks of Excessive N	Reference
Antioxidant enzyme system	SOD	Activity significantly enhanced in sunflower under 80–120 kg N ha^−1^; O_2_^−^ scavenging efficiency improved	Induced secondary oxidative burst; exacerbated membrane damage	[54]
	CAT	Activity enhanced in cotton under 5 mM N; H_2_O_2_ content reduced to 55% of low-N drought groups	Carbon-N metabolic imbalance; increased respiratory load	[55]
	APX	Synergized with GR to maintain ascorbate–glutathione cycle stability	Accumulation of photorespiratory H_2_O_2_	
Osmotic Adjustment	Proline	Synthesis increased by 24% in maize under 180 kg N ha^−1^; activated via P5CS pathway	Metabolic disorder due to overaccumulation	[56]
	Soluble sugars (sucrose, fructans)	Content increased by 30% in wheat under 0.6 g N kg^−1^ soil; enhanced cellular osmolarity and membrane stability	Uncoupling from N assimilation; toxic soluble sugar accumulation	[57]
Structural reinforcement	Xylem vessels and sclerenchyma	Xylem vessel diameter increased by 18% and sclerenchyma thickness by 25% in wheat; reduced drought-induced cavitation	Promoted succulent growth; increased water demand	[57]

**Table 3:** Root architectural responses to N levels in plants.

N Level	Root Structural Responses	Implications for Drought Adaptation	Representative Crop	Reference
Moderate N	Increased deep root proportion (30–50% higher root length density in >40 cm soil layers); proliferation of fine roots (<0.5 mm diameter)	Enhanced deep soil water uptake; improved hydraulic conductance; increased drought survival rate	Maize (*Zea mays)*	[68]
	Radial expansion of xylem vessels; synergized with fine root proliferation to optimize water transport efficiency	Improved root-to-canopy water transport; maintained transpiration-photosynthesis balance	*Catalpa bungei*	[28]
N deficiency	Increased shallow fine root density; prioritized carbon allocation to roots for expanded absorption area	Enhanced surface soil resource capture, but insufficient deep water acquisition	Maize (*Zea mays*)	[66]
High N supply	Shallow root distribution; reduced R/S; decreased total root biomass	Rapid depletion of surface soil moisture; increased hydraulic vulnerability under drought	Winter wheat (*Triticum aestivum*)	[30]
N form difference	Preferential NH_4_^+^ uptake in *Malus prunifolia* under drought; upregulated AMT1;2/4;2 transporter expression	Conserved energy consumption; maintained nodule vitality under N-limited drought stress	Apple rootstock (*Malus prunifolia*)	[31]

## Data Availability

Not applicable.
